# Multifunctional Bioactivity of Halolactones Derived from Vanillin and Their Effects on Lipid Membranes: Biological and Biophysical Evaluation

**DOI:** 10.3390/ijms27114821

**Published:** 2026-05-27

**Authors:** Anna Dunal, Aleksandra Włoch, Dominik Poradowski, Aleksander Chrószcz, Witold Gładkowski, Hanna Pruchnik

**Affiliations:** 1 Department of Food Chemistry and Biocatalysis, Faculty of Biotechnology and Food Sciences, Wrocław University of Environmental and Life Sciences, Norwida 25, 50-375 Wrocław, Poland; witold.gladkowski@upwr.edu.pl; 2 Department of Physics and Biophysics, Faculty of Biotechnology and Food Sciences, Wrocław University of Environmental and Life Sciences, Norwida 25, 50-375 Wrocław, Poland; aleksandra.wloch@upwr.edu.pl (A.W.); hanna.pruchnik@upwr.edu.pl (H.P.); 3 Department of Biostructure and Animal Physiology, Division of Animal Anatomy, Faculty of Veterinary Medicine, Wroclaw University of Environmental and Life Sciences, Kożuchowska 1, 51-631 Wrocław, Poland; dominik.poradowski@upwr.edu.pl (D.P.); aleksander.chroszcz@upwr.edu.pl (A.C.)

**Keywords:** vanillin, halolactones, antiproliferative activity, antioxidant activity, human erythrocyte, lipid organization, model membrane, spectroscopy

## Abstract

A series of novel vanillin-derived halolactones bearing a phenolic ring at the β-position were comprehensively evaluated for their antioxidant and anti-inflammatory properties. The most active derivative, *trans*-4-(4′-hydroxy-3′-methoxyphenyl)-5-(1-iodoethyl)dihydrofuran-2(3H)-one (**LV2**), exhibited strong antioxidant activity in the ABTS assay (EC_50_ = 35.65 ± 2.34 μM), comparable with Trolox^®^ and superior to the reference compound in the UV-C-induced oxidative stress assay (IC_50_ = 48.67 ± 5.46 μM). Among the tested lactones, **LV2** showed moderate anti-inflammatory activity in the COX-1 and COX-2 inhibition assays. Further studies revealed that iodolactone **LV2** exhibited high antiproliferative activity in the MTT assay, particularly against the LM-MEL-75 and EPG85-257RDB cell lines (IC_50_ = 8.67 ± 10.61 μM and 7.37 ± 3.23 μM, respectively), along with high selectivity (selectivity indices > 5). The effects of iodolactone **LV2** on various lipid membranes were examined using fluorometric methods, while its impact on human red blood cell morphology was evaluated by analyzing erythrocyte shape changes. The influence of **LV2** on model membrane organization was further investigated using attenuated total reflectance-Fourier transform infrared spectroscopy (ATR-FTIR). All spectroscopic studies confirmed the lactone’s interaction with polar regions of model membranes, demonstrating its capacity to modulate membrane-associated functions. This multifunctional bioactivity positions iodolactone **LV2** as a promising candidate for further anticancer studies.

## 1. Introduction

According to the World Health Organization (WHO), cancer remains one of the leading causes of mortality worldwide, accounting for nearly 10 million deaths annually. Moreover, its incidence continues to rise, and projections indicate that by 2040, approximately 30 million new cancer cases will be diagnosed globally, representing about a 57% increase compared with 2020 [1].

Numerous studies have demonstrated that oxidative stress strongly influences carcinogenesis. In particular, an imbalance between reactive oxygen species (ROS) production and the body’s antioxidant defenses plays a crucial role in cancer initiation and progression [2,3,4,5]. Excessive ROS levels induce oxidative damage to key cellular macromolecules, including DNA, proteins, and lipids, thereby promoting genomic instability and the dysregulation of signaling pathways involved in cancer development [6,7]. Oxidative stress also contributes to chronic inflammation and tissue damage, creating a cancer-promoting microenvironment [8,9]. Contemporary research increasingly focuses on bioactive compounds that reduce oxidative damage, modulate inflammatory responses and selectively target cancer cells. To better understand the mechanisms of anticancer compounds, it is therefore essential to investigate not only their antioxidative and anti-inflammatory properties, but also their interactions with biological membranes, which play a crucial role in maintaining cellular function and integrity. Membrane fluidity, in particular, is a crucial factor for maintaining proper cellular processes and protecting healthy cells, as variations in fluidity influence both normal physiological functions and disease pathophysiology [10,11].

In cancer cells, increased membrane fluidity is closely associated with enhanced invasiveness, proliferation, and metastatic potential [12]. Studying how different compounds affect the physical properties of cancer cell membranes, including fluidity, rigidity and lipid organization, can provide essential insights into their cytotoxic mechanisms and therapeutic actions [13,14]. For numerous anticancer agents, direct interactions with the cell membrane represent a key determinant of their effectiveness [15,16]. Understanding these membrane-related differences not only helps identify selective anticancer strategies but also offers approaches for designing therapies that protect normal tissues while efficiently targeting cancer cells. Among others, lactones have emerged as particularly promising bioactive compounds due to their broad spectrum of biological activities [17,18,19]. This large, structurally diverse group of compounds is commonly found as secondary metabolites in plants [20,21,22], microorganisms [23,24], insects [25,26], and marine organisms [27,28]. As natural products, they are recognized as valuable sources for discovering and developing novel bioactive compounds [29,30]. A broad spectrum of activities, including antibacterial [31,32,33], antifungal [34,35], antioxidant [36,37], anti-inflammatory [38,39], antifeedant [40,41,42], antiviral [43,44], and anticancer [45,46,47,48,49] makes them important compounds in medicine, veterinary medicine, pharmacy, cosmetology, agriculture and the food industry. In particular, lactones bearing aromatic substituents have attracted considerable attention due to their pronounced antiproliferative activity [50,51,52,53].

In our previous study, we obtained a series of novel γ-halo-δ-lactones and δ-halo-γ-lactones bearing a phenolic ring at the β-position via a 7-step synthesis starting from vanillin (**V**). These halolactones exhibited antiproliferative activity against two canine (CLBL-1, CLB70) and two human (T-24, Caco-2) cancer cell lines. The most active compound was *trans*-4-(4′-hydroxy-3′-methoxyphenyl)-5-(1-iodoethyl)dihydrofuran-2(3H)-one, hereafter referred to as **LV2**. None of the tested compounds showed toxicity against human erythrocytes and the normal mouse embryonic fibroblast cell line (NIH/3T3) [54]. Among the synthesized lactones, **LV2** exhibited the highest antiproliferative activity. Combined with its lack of toxicity against normal cells, this makes it a particularly suitable candidate for further biological and biophysical studies.

However, despite growing interest in halolactones, their biological activity has been evaluated mainly in terms of cytotoxicity, while their antioxidant and anti-inflammatory potential remain insufficiently characterized. Importantly, their interactions with biological membranes—critical determinants of cellular response, selectivity and mechanism of action—have largely been overlooked. This lack of multifunctional evaluation represents a significant gap in understanding their biological and membrane-related properties.

The present study addresses the need for an in-depth investigation of vanillin-derived halolactones. Continuing our previous research, we focused on a comprehensive evaluation of their antioxidant and anti-inflammatory activities. For the most active compound (**LV2**), we report the antiproliferative assay results against a panel of human cancer cell lines, as well as its effects on biological and model membranes, including its ability to induce morphological changes in human erythrocytes.

## 2. Results and Discussion

### 2.1. Studied Halolactones

**Figure 1:**
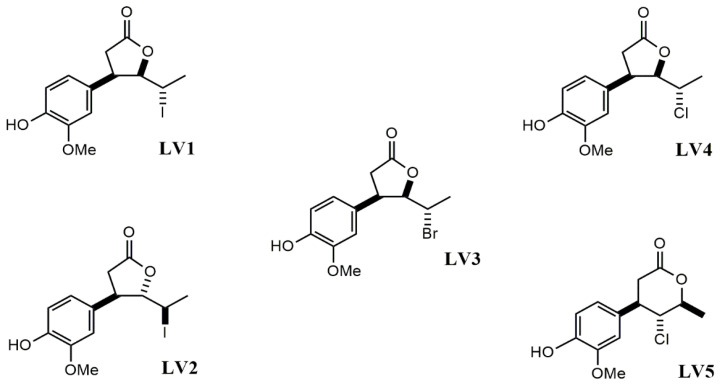
Structures of the tested halolactones: *cis*-4-(4′-hydroxy-3′-methoxyphenyl)-5-(1-iodoethyl)dihydrofuran-2-one (**LV1**), *trans*-4-(4′-hydroxy-3′-methoxyphenyl)-5-(1-iodoethyl)dihydrofuran-2-one (**LV2**), *cis*-5-(1-bromoethyl)-4-(4′-hydroxy-3′-methoxyphenyl)dihydrofuran-2-one (**LV3**), *cis*-5-(1-chloroethyl)-4-(4′-hydroxy-3′-methoxyphenyl)dihydrofuran-2-one (**LV4**), and 5-*t*-chloro-4-*r*-(4′-hydroxy-3′-methoxyphenyl)-6-*c*-methyltetrahydropyran-2-one (**LV5**).

The study focused on a series of novel vanillin-derived γ-halo-δ-lactones and δ-halo-γ-lactones bearing a phenolic ring at the β-position (Figure 1).

These compounds (**LV1**–**LV5**) were synthesized from vanillin (**V**) through a seven-step synthetic pathway. The synthetic procedure was described in detail in our previous publication [54] and a scheme presenting the synthetic pathway, including reagents and reaction conditions, is provided in the Supplementary Materials (Scheme S1).

### 2.2. Biological Research

#### 2.2.1. Free-Radical Scavenging Assays and Thiobarbituric Acid Reactive Substances Assay (TBARS Assay)

The antioxidant activity of the tested lactones (**LV1**–**LV5**) was evaluated and compared with that of the starting compound, vanillin (**V**), against two free radicals: DPPH^•^ (2,2-diphenyl-1-picrylhydrazyl radical) and ABTS^•+^ (2,2′-azobis(3-ethylbenzothiazoline-6-sulfonic acid cation radical)), using spectrophotometric methods. In this study, Trolox^®^ was used as the standard reference for antioxidant activity assays [55]. The calculated values of EC_50_ for the analyzed compounds are shown in Table 1.

Vanillin (**V**) exhibited very low radical scavenging activity. In the DPPH assay, no activity was detected at the tested concentrations, and very low activity was observed against the ABTS^•+^ radical. This finding indicates the limited antioxidant properties of the starting compound.

Chemical transformation of vanillin (**V**) into halolactones **LV1**–**LV5** markedly enhanced antioxidant activity, particularly in the ABTS assay. Among the synthesized compounds, the most active was the *trans* δ-iodo-γ-lactone (**LV2**) (EC_50_ = 293.82 µM in the DPPH^•^, EC_50_ = 35.67 µM in the ABTS^•+^). Its activity against ABTS^•+^ was comparable to that of Trolox^®^ (EC_50_ = 31.42 µM), indicating its strong radical scavenging potential. A similar improvement in radical-scavenging activity following chemical modification has also been observed for structurally related phenolic lactones, such as coumarins, where derivatization led to improved antioxidant properties through the electronic redistribution and stabilization of radical intermediates [56].

To compare the antioxidant activity of vanillin (**V**) and halolactones **LV1**–**LV5** against a phosphatidylcholine model lipid membrane (PLM), the TBARS assay was performed using UV-C radiation to induce oxidative stress. The extent of lipid peroxidation in the model PLM membrane was determined spectrophotometrically by measuring malondialdehyde (MDA) formation. The results of this assay are presented in Table 2.

Vanillin (**V**) exhibited very low activity (IC_50_ > 800 µM), whereas for all vanillin-derived halolactones (**LV1**–**LV5**), marked inhibition of UV-C-induced oxidation was observed, confirming that the transformation of vanillin into halolactones markedly enhances the antioxidant potential of these compounds.

Based on the results of the antioxidant assays, conclusions regarding the relationship between the structure of the tested halolactones and their biological activity can be drawn. Comparison of the *cis* δ-iodo-γ-lactone (**LV1**) and *trans* δ-iodo-γ-lactone (**LV2**) revealed a pronounced influence of stereochemistry on antioxidant activity, as demonstrated in the free radical scavenging assays. The *trans*-isomer was significantly more active (EC_50_ = 293.82 µM for DPPH^•^, EC_50_ = 35.67 µM for ABTS^•+^) than the *cis* isomer (EC_50_ = 810.53 µM for DPPH^•^, EC_50_ = 125.88 µM for ABTS^•+^), indicating that the spatial arrangement plays a crucial role in radical neutralization efficiency. Similarly, in the TBARS assay, the *trans* isomer exhibited significantly stronger inhibition of UV-C-induced oxidation of the PLM (IC_50_ = 48.67 µM) than the *cis* isomer **LV1** (IC_50_ = 97.21 µM). The activity of iodolactone **LV2** was comparable to that of Trolox^®^ (IC_50_ = 64.56 µM), used as a positive control. A clear effect of stereochemistry on the antioxidant activity was also observed for hydroxystilbene derivatives. Isomers with the *trans* configuration of the double bond typically exhibit higher radical scavenging activity than those with a *cis* configuration, due to lower O-H bond dissociation enthalpies and more efficient delocalization of unpaired electrons, thereby facilitating hydrogen atom transfer processes [57].

Within the group of halolactones containing different halogen atoms, the *cis* δ-bromo-γ-lactone (**LV3**) demonstrated higher activity (EC_50_ = 80.44 µM) than its corresponding structural analogs, the *cis* δ-chloro-γ-lactone (**LV4**, EC_50_ = 129.95 µM), and the *cis* δ-iodo-γ-lactone (**LV1**, EC_50_ = 125.88 µM) in the ABTS assay. Among all of the tested compounds, the lowest activity was found for γ-chloro-δ-lactone (**LV5**). In the TBARS assay, the results revealed the following halogen-dependent trend: iodolactones > bromolactone > chlorolactones. The *cis* δ-iodo-γ-lactone **LV1** showed higher activity than the *cis*-δ-bromo-γ-lactone **LV3** (IC_50_ = 97.21 µM *versus* IC_50_ = 146.7 µM). The weakest effects were observed for the *cis*-δ-chloro-γ-lactone **LV4** (IC_50_ = 545.98 µM) and the γ-chloro-γ-lactone **LV5** (IC_50_ = 663.27 µM).

Taken together, the antioxidant assays consistently indicate a clear structure–activity relationship, in which both stereochemistry and halogen substitution strongly influence the antioxidant and antiperoxidative activity of vanillin-derived halolactones. In particular, the *trans* configuration of substituents at the γ-lactone ring and the presence of iodine appear to be key determinants of the highest biological activity observed for iodolactone **LV2**, which acts as a potent scavenger of ABTS^•+^ radicals and a highly active inhibitor of UV-C-induced lipid peroxidation in the PLM, suggesting strong protection against oxidative membrane damage.

#### 2.2.2. Anti-Inflammatory Activity

To evaluate the anti-inflammatory activity of the vanillin-derived halolactones, enzymatic assays were performed using cyclooxygenase-1 (COX-1) and cyclooxygenase-2 (COX-2) as model targets. Among the tested compounds, only the *trans* isomer of iodolactone **LV2** exhibited measurable inhibitory activity, whereas the remaining derivatives showed no significant effect. The results are presented in Table 3.

The Student’s *t*-test revealed statistically significant differences between the tested groups in both COX-1 and COX-2 activities (*p* < 0.05). The iodolactone **LV2** exhibited moderate inhibition of both COX isoforms. The reference anti-inflammatory drug ibuprofen demonstrated much stronger inhibition, particularly against COX-2, highlighting that iodolactone **LV2** exhibits only limited anti-inflammatory activity and suggesting a limited impact on COX-mediated pathways under the tested conditions.

Based on the results of the antioxidant and anti-inflammatory activity studies, as well as the data on the antiproliferative activity and toxicity against human erythrocytes reported in our earlier publication [54], the most active compound, the *trans*-isomer of iodolactone **LV2**, was selected for further biological and biophysical investigations.

#### 2.2.3. Antiproliferative Activity

To expand upon previous studies of its antiproliferative potential, the vanillin-derived *trans*-iodolactone **LV2** was evaluated in an in vitro MTT assay against a panel of human cancer cell lines. This panel was selected to provide a broad representation of cancer types, including human lung adenocarcinoma (A549), breast carcinoma (MCF-7), melanoma (LM-MEL-75), cervical carcinoma (HeLa), osteosarcoma (Saos-2), ovarian carcinoma (A2780), and gastric adenocarcinoma (EPG85-257RDB). For comparison, normal human dermal fibroblasts (NHDFs) were also included as a representative non-cancerous cell line. The MTT assay results for iodolactone **LV2** were compared with those of the parent vanillin (**V**) and doxorubicin as a positive control (Table 4).

Vanillin (**V**) demonstrated measurable activity against three cancer cell lines: A549, EPG85-257RDB, and LM-MEL-75. No significant antiproliferative activity was observed against the remaining cell lines.

Structural modification of vanillin (**V**) to the *trans*-iodolactone **LV2** resulted in altered antiproliferative activity against the cancer cell lines, with improvements observed in some cases. The most pronounced effects were observed for the LM-MEL-75 cell line (IC_50_ = 8.67 µM compared with 36.28 µM for vanillin), the EPG85-257RDB cell line (IC_50_ = 7.37 µM compared with 33.65 µM for vanillin), and the A2780 cell line (IC_50_ = 13.62 µM compared with >50.00 µM for vanillin). A further increase in activity was also detected for the Saos-2 cell line (IC_50_ = 37.90 µM compared with >50.00 µM for vanillin). For the A549 cell line, iodolactone **LV2** exhibited slightly lower activity than vanillin (IC_50_ = 33.92 µM versus 24.52 µM). No activity of iodolactone **LV2** was observed against MCF-7 breast cancer cells or HeLa cells (IC_50_ > 50 µM). Notably, the *trans*-isomer of iodolactone **LV2** did not show cytotoxicity against the normal NHDF cell line (IC_50_ > 50 µM).

To quantitatively assess the preference of the tested compounds for cancer cells over normal cells, the selectivity index (SI) was calculated as the ratio of the IC_50_ value determined for NHDFs to that obtained for a given cancer cell line. SI values greater than 1 indicate selective cytotoxicity toward cancer cells [58] (Table 5).

As shown in Table 5, vanillin (**V**) exhibited SI values above 1 in three tested cell lines. Overall, the relatively low SI values indicate that vanillin (**V**) displays limited cancer cell selectivity, consistent with its weak antiproliferative potency. In contrast, iodolactone **LV2** showed higher selectivity indices, particularly against the EPG85-257RDB (SI > 6.78), LM-MEL-75 (SI > 5.77), and A2780 (SI > 3.67) cell lines. These results indicate a clear preference of iodolactone **LV2** for selected cancer cell lines, particularly those of melanoma and gastric adenocarcinoma origin. Lower SI values, but still >1, were observed for A549 and Saos-2 reflecting limited selectivity toward these cell lines. Overall, these findings demonstrate that the conversion of vanillin (**V**) into the corresponding *trans*-iodolactone **LV2** significantly improves antiproliferative activity and enhances cancer cell selectivity in selected cell lines.

Doxorubicin, used as a reference anticancer drug, exhibited very strong antiproliferative activity against all tested cancer cell lines, with IC_50_ values in the submicromolar range (approximately 0.68–5.48 µM). However, its high cytotoxicity was also evident against NHDF cells (IC_50_ = 0.44 µM), confirming its well-documented lack of selectivity toward cancer cells and explaining the severe side effects associated with its clinical use. Consequently, doxorubicin displayed SI values < 1 for all tested cell lines, indicating a lack of cancer cell selectivity and highlighting its well-known dose-limiting toxicity and severe side effects.

The antiproliferative activity profile of the *trans*-iodolactone **LV2** observed in the present study agrees well with our previous studies on vanillin-derived halolactones. In an earlier report, the corresponding *trans* δ-iodo-γ-lactone (**LV2**) exhibited the highest activity among the tested analogues against hematopoietic canine cancer cell lines, particularly CLBL-1 (IC_50_ = 46.3 µM), while remaining non-toxic to normal NIH/3T3 fibroblasts [54]. In comparison, the current results demonstrate markedly enhanced potency of iodolactone **LV2** against selected human solid tumor cell lines, with IC_50_ values in the low micromolar range (7.37–13.62 µM for EPG85-257RDB, LM-MEL-75, and A2780 cells). This improvement suggests that the *trans*-iodolactone (**LV2**) structure derived from vanillin (**V**) may be particularly effective against specific human cancer types, including melanoma and gastric adenocarcinoma. Importantly, the *trans*-isomer of iodolactone **LV2** retained a favorable selectivity profile in both studies: no cytotoxicity against normal mouse fibroblasts in the previous study and the high SI values observed in the present study. These findings underscore the benefit of the *trans*-iodolactone (**LV2**) modification in enhancing both antiproliferative potency and cancer cell selectivity, key aspects in cytostatic drug development.

Structural analogues of iodolactone **LV2**, including halolactones synthesized from simple aromatic aldehydes like 2,5-dimethylbenzaldehyde, piperonal or cuminaldehyde have also demonstrated notable antiproliferative activity. In previous studies, these compounds exhibited cytotoxic effects against a range of cancer cell lines, including Jurkat, GL-1, CLBL-1, D17, and CLB70. These findings suggest that the structural modification of aromatic aldehydes into halolactone derivatives represents an effective strategy for enhancing antiproliferative properties [59,60,61].

### 2.3. Biophysical Research

Biological membranes play a critical role in cellular function and in mediating responses to bioactive compounds, with lipid organization and membrane fluidity influencing both drug uptake and cytotoxicity. Investigating the effects of halolactones on membranes formed from lipids isolated from erythrocytes (LEMs), erythrocyte membranes (RBCMs), and mimic cancer cell lipid bilayers (MCLMs) can therefore provide insights into their mechanisms of action and selectivity.

#### 2.3.1. Shape of Red Blood Cells (RBCs)

RBCs represent a sensitive in vitro model for evaluating compound interactions with cellular membranes and overall cytotoxicity. Morphological alterations in erythrocytes, including echinocytic (E) and stomatocytic (S) transformations, reflect changes in membrane integrity and can serve as indicators of compound–membrane interactions.

**Figure 2:**
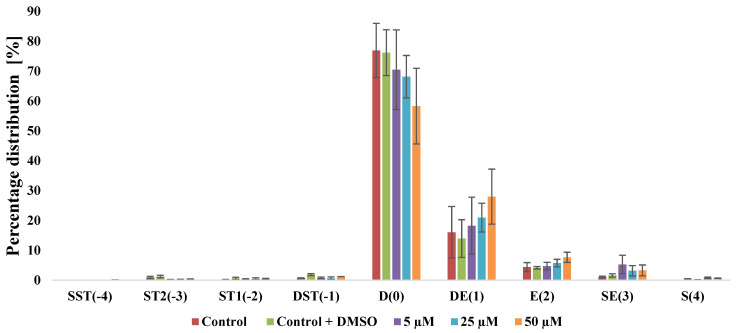
Percentage distribution of erythrocyte morphological forms induced by iodolactone **LV2**. The names of the shapes and their abbreviations (used in the figure) are as follows: spherostomatocytes (SST(-4)), second-order stomatocytes (ST2(-3)), first-order stomatocytes (ST1(-2)), discostomatocytes (DST(-1)), discocytes (D(0)), discoechinocytes (DE(1)), echinocytes (E(2)), spheroechinocytes (SE(3)), and spherocytes (S(4)). Values are presented as means ± SD (n = 3).

To evaluate the effect of the vanillin-derived iodolactone **LV2** on human erythrocyte morphology, we analyzed shape changes in erythrocytes exposed to increasing concentrations of the compound. Three major erythrocyte shapes—discocytes (D), stomatocytes (S), and echinocytes (E)—were identified and quantified by optical microscopy, with the proportion of each shape calculated as a percentage of the total cells examined. The results, presented in Figure 2, show the relative distribution of erythrocyte shapes induced during iodolactone **LV2** treatment.

Statistical analysis was performed using one-way analysis of variance (ANOVA), followed by Dunnett’s multiple comparison test for comparisons with the control group.

Incubation of human erythrocytes with iodolactone **LV2** resulted in concentration-dependent morphological changes. Statistical significance relative to the control group is presented in Figure S1A–I in the Supplementary Materials. In control samples, discocytes (D(0)) predominated, accounting for approximately 77% of the erythrocyte population (76.92% in the untreated control and 76.20% in the vehicle control containing DMSO). Upon iodolactone **LV2** treatment, the proportion of discocytes decreased progressively with increasing compound concentration, reaching 70.45% at 5 μM, 68.13% at 25 μM, and 58.30% at 50 μM. Concurrently, echinocytic forms increased. Discoechinocytes (DE(1)) rose from 16.03% (control) to 18.25%, 20.96%, and 27.98% at 5, 25, and 50 μM, respectively. A similar trend was noted for echinocytes (E(2)), whose proportion increased from 4.40% in the control to 4.72%, 5.71%, and 7.65% with increasing iodolactone **LV2** concentrations. Spheroechinocytes (SE(3)) showed slight increases: 5.28% at 5 μM and stabilized at 3.16–3.29% at higher concentrations. Other erythrocytes were observed only sporadically.

In summary, exposure to vanillin-derived iodolactone **LV2** resulted in concentration-dependent trends toward echinocytic transformation in human erythrocytes. Morphological analysis also demonstrated a decrease in discocytes accompanied by an increase in echinocyte populations (Figure 2). However, statistically significant differences compared with the control were observed only for selected morphological forms, including an increase in second-order stomatocytes (ST2(-3)) at 5 and 25 µM and a decrease in spherocytes (S(4)) at 25 and 50 µM, with a minor but significant effect also observed for the DMSO control. The observed pattern suggests a potential concentration-dependent effect of **LV2** on erythrocyte morphology. According to the Sheetz–Singer model, this effect results from compounds preferentially incorporating into the outer lipid monolayer, thereby expanding it relative to the inner layer and causing outward membrane deformation with characteristic spicules [62].

The above findings suggest that iodolactone **LV2** associates primarily with the outer leaflet of the tested membrane. The observed increase in both the number and transformation stage of echinocytes with rising compound concentrations indicates that its effects likely result from interactions predominantly at the external monolayer of the cell membrane.

#### 2.3.2. Fluorometric Methods

To evaluate the influence of iodolactone **LV2** on membrane physicochemical properties, fluorescence spectroscopy experiments were performed using four complementary probes: MC540 (Merocyanine 540), Laurdan (1-[6-(dimethylamino)naphthalen-2-yl]dodecan-1-one), DPH (1,6-diphenyl-1,3,5-hexatriene), and TMA-DPH (*N*,*N*,*N*-trimethyl-4-(6-phenyl-1,3,5-hexatrien-1-yl)phenylammonium *p*-toluenesulfonate) in three model membrane types: erythrocyte lipid extract-based membranes (LEMs), intact erythrocyte membranes (RBCMs), and cancer-mimicking lipid membranes (MCLMs).

The MCLMs were designed and composed in this study to reflect the average lipid profiles of cancer cell lines [63,64,65,66,67]. This approach was chosen because the iodolactone **LV2** showed the strongest antiproliferative activity and selectivity against three cancer cell lines (LM-MEL-75, A2780, and EPG85-257RDB), suggesting that membrane-related effects may contribute to its mechanism of action.

The results obtained using the MC540 probe are presented in Table 6 as percentage changes in fluorescence intensity relative to the control.

Statistical analysis was performed using one-way analysis of variance (ANOVA), followed by Dunnett’s multiple comparison test for comparisons with the control group.

The probe MC540 localizes near the membrane surface, specifically in the outer leaflet and is highly sensitive to lipid packing and polarity in the bilayer and the surrounding aqueous environment [68,69]. As a heterocyclic chromophore with a localized negative charge, the MC540 probe selectively binds to the glycerol-backbone region of the outer phospholipid leaflet, with the extent of binding depending on membrane packing density [70]. Its fluorescence intensity increases in the liquid phase of lipid bilayers and decreases in the gel phase. MC540 fluorescence was used to assess disturbances in the polar region of membranes and lipid packing. Statistical significance relative to the control group is presented in Figure S2A,B in the Supplementary Materials. In LEMs, iodolactone **LV2** induced a statistically significant increase in fluorescence intensity relative to the control at all tested concentrations, reaching approximately 23% relative to the control at 30–40 µM, indicative of perturbations in lipid packing within the polar headgroup region. A similar, statistically significant increase in fluorescence intensity relative to the control was observed in RBCMs, with fluorescence rising by nearly 77% relative to the control at 30 µM, indicating pronounced changes in membrane surface organization rather than minor alterations. MCLMs exhibited higher baseline fluorescence, which was significantly affected by iodolactone **LV2**, showing predominantly moderate, concentration-dependent increases and reflecting altered lipid organization in cancer-mimicking membranes (Table 6).

**Figure 3:**
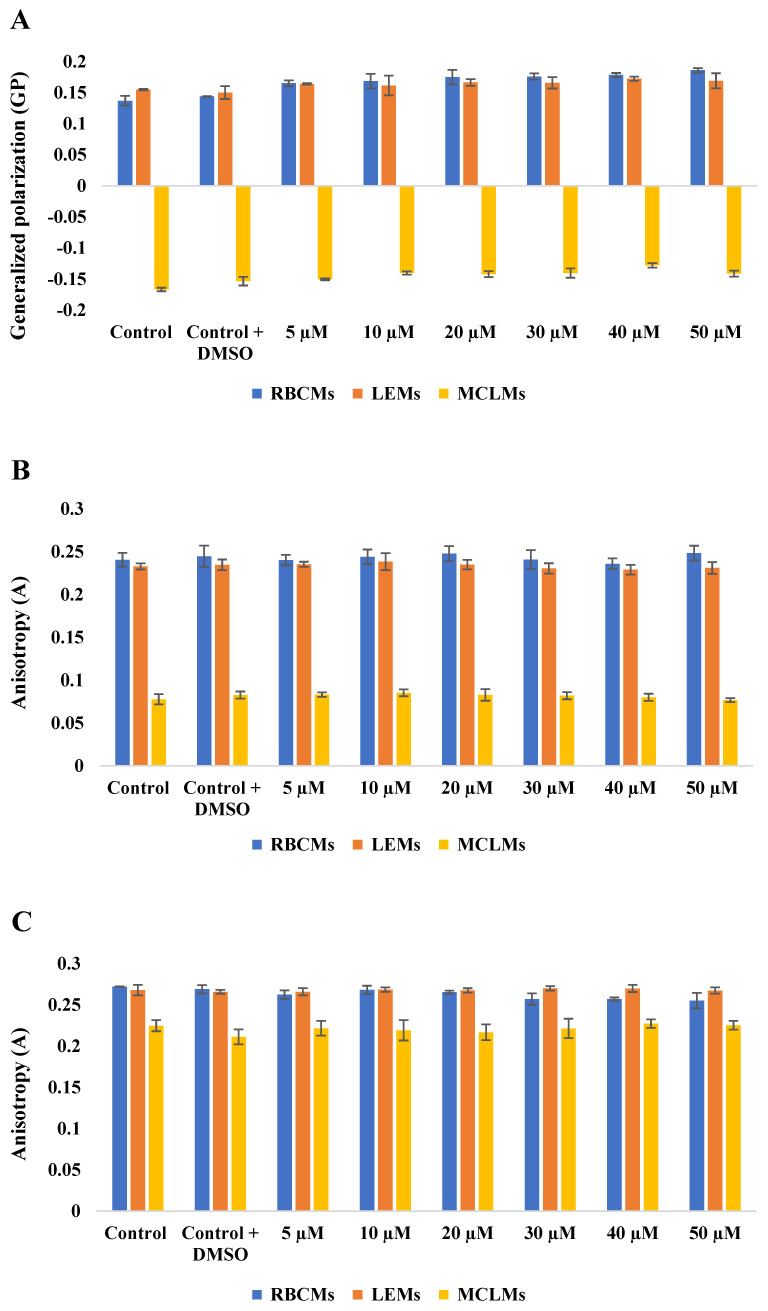
Generalized polarization of Laurdan (**A**) and fluorescence anisotropy of DPH (**B**) and TMA-DPH (**C**) in LEMs, RBCMs, and MCLMs in the presence of iodolactone **LV2**. Values are shown as mean ± SD (n = 3). Statistical analysis was performed using one-way analysis of variance (ANOVA), followed by Dunnett’s multiple comparison test for comparisons with the control group. Analyses were performed separately for each model membrane.

Further membrane interactions of iodolactone **LV2** were assessed using Laurdan generalized polarization (GP) values, while DPH and TMA-DPH fluorescence anisotropy (A) provided complementary information on hydrocarbon chain fluidity in phospholipid bilayers. Data for these three probes are shown in Figure 3A–C.

It should be noted that MC540 and Laurdan probe different regions and physicochemical properties of the membrane; therefore, they may reveal seemingly different but complementary effects. Laurdan, due to its twelve-carbon aliphatic chain, embeds itself in the nonpolar interior of the bilayer, while its fluorescent group is positioned at the level of the lipid ester groups. This makes it sensitive to polarity changes and capable of reporting on hydration and packing order within the lipid headgroups of LEMs, RBCMs and MCLMs [71,72,73]. Generalized polarization (GP) of membrane-incorporated Laurdan provides complementary information on membrane packing, polarity and hydration [74]. Higher GP values indicate increased lipid order and reduced water penetration, whereas lower values reflect more disordered, fluid membranes. Statistical significance relative to the control group is presented in Figure S3A,B. In LEMs, GP values increased in a statistically significant manner at multiple concentrations, rising from 0.16 (control) to 0.17 at 40 µM, indicating a statistically confirmed increase in lipid order in the interfacial region of the membrane. RBCMs showed a more pronounced trend, with GP rising from 0.14 (control) to 0.19 at 50 µM of **LV2**, reflecting significant progressive membrane rigidification at all concentrations tested at the interface region. In contrast, MCLMs showed strongly negative GP values (from −0.17 in the control to −0.13 at 40 µM of **LV2**), consistent with a highly disordered and hydrated lipid environment characteristic of cancer-mimicking membranes. **LV2** treatment caused a partially significant shift toward less negative GP values, with statistical significance observed for most concentrations (*p* < 0.05–0.0001), while the DMSO control also showed a small but statistically significant effect (*p* = 0.019). This indicates that even the vehicle contributes to baseline membrane perturbation and should be considered in the interpretation. The apparent discrepancy between increased MC540 fluorescence and elevated Laurdan GP values can be rationalized by membrane lateral heterogeneity, where **LV2** induces the coexistence of ordered domains and localized packing defects within the same membrane system. This effect may contribute to the selective antiproliferative activity of iodolactone **LV2** against melanoma, ovarian carcinoma, and gastric adenocarcinoma cells (Figure 3A).

DPH was employed to assess the properties of the hydrophobic core of the phospholipid bilayers. Due to its nonpolar nature, DPH embeds within the fatty acyl chain region, providing information on the order and dynamics of the membrane’s hydrophobic interior. TMA-DPH was used to evaluate the properties of the interfacial region of the phospholipid bilayers. Its cationic trimethylammonium group anchors the probe near the lipid headgroup region, while the hydrophobic DPH moiety embeds partially into the acyl chains. This arrangement allows TMA-DPH to report on the order and dynamics of the membrane’s interfacial region [75]. The effects of iodolactone **LV2** on membrane dynamics were assessed using the fluorescence anisotropy (A) of DPH and TMA-DPH probes. Lower anisotropy (A) indicates increased membrane fluidity, whereas higher values correspond to greater lipid ordering and rigidity [76].

For the DPH probe, no statistically significant differences compared with the control were observed in either LEMs or RBCMs across all tested concentrations (Figure S4B). Anisotropy values remained close to the control levels (LEMs 0.23–0.24; RBCMs 0.24–0.25), indicating that iodolactone **LV2** did not substantially affect the hydrophobic core fluidity of normal erythrocyte-derived membranes. In contrast, MCLMs exhibited significantly lower baseline DPH anisotropy (0.08–0.09) compared with normal cell membranes (erythrocytes), consistent with their intrinsically more fluid lipid composition. Notably, iodolactone **LV2** induced a slight but detectable concentration-dependent increase in anisotropy, suggesting subtle lipid ordering and mild membrane rigidification in MCLMs (Figure 3B).

Similar effects were observed with the TMA-DPH probe, which localizes in the hydrophilic–hydrophobic interfacial region and reports on the dynamics above the hydrocarbon chains. LEMs and RBCMs displayed anisotropy values close to the control levels, with a statistically significant decrease observed only at selected higher concentrations (30–50 µM, depending on the membrane type), suggesting a modest fluidizing effect at the interface (Figure S5A,B). MCLMs showed lower anisotropy values (ranging from 0.21 to 0.23), but iodolactone **LV2** induced minor changes consistent with subtle ordering effects in the upper acyl chain region of the bilayer. Importantly, the distinct baseline properties of the MCLMs membranes, characterized by lower anisotropy, confirm their more fluid and disordered nature compared with normal erythrocyte membranes. The slight ordering induced by iodolactone **LV2** in this cancer-mimicking system may therefore contribute to its selective antiproliferative activity against cancer cells with altered lipid composition (Figure 3C).

Overall, the anisotropy measurements indicate that iodolactone **LV2** exerts concentration-dependent but generally modest effects on membrane fluidity, with RBCMs remaining largely unaffected while MCLMs showed slight membrane rigidification consistent with partial lipid ordering. The observed changes in DPH anisotropy, combined with the more pronounced effects detected by Laurdan, MC540, and TMA-DPH, suggest that iodolactone **LV2** primarily interacts with the membrane’s interfacial region, rather than uniformly affecting the entire bilayer structure or deeply penetrating the hydrophobic core. Similar membrane-related effects were previously reported for bromolactones bearing 2,5-dimethylphenyl substituents. Fluorescence spectroscopy showed that these compounds preferentially accumulated in the hydrophilic interfacial region of erythrocyte membranes, affecting lipid headgroup packing while minimally influencing the hydrophobic core, as reflected by only minimal changes in DPH anisotropy that preserved acyl chain fluidity [60]. Włoch et al. reported that, in cancer cell lines (GL-1 and Jurkat) the (-)-(4*S*,5*R*,6*S*) enantiomer of piperonal-derived *trans* β-aryl-δ-iodo-γ-lactones exerted a more pronounced effect on membrane fluidity than the (+)-(4*R*,5S,6*R*) enantiomer. Notably, in their mimic model membrane (MIMIC), no significant anisotropy changes were observed, suggesting that membrane disturbances in cancer cells may be driven largely by membrane proteins rather than by lipid-only interactions. In our study, however, slight but detectable anisotropy changes were observed even in the cancer-mimicking system. This discrepancy may arise from differences in membrane composition. The MCLMs used in this study were specifically designed to mimic the average lipid profiles of melanoma, ovarian carcinoma, and gastric adenocarcinoma cells, which may confer distinct baseline fluidity and packing properties compared with the MIMIC model used by Włoch et al. The racemic nature of iodolactone **LV2** (as opposed to a single enantiomer) may further contribute to the observed differences [77]. Moreover, the absence of proteins in lipid-only models can increase the sensitivity of fluorescence probes to subtle ordering effects induced directly by iodolactone **LV2**. Therefore, even the minor rigidification observed in MCLMs may reflect direct lipid–compound interactions that could contribute to the selective antiproliferative activity of iodolactone **LV2** against cancer cells with altered lipid organization.

#### 2.3.3. Attenuated Total Reflectance-Fourier Transform Infrared Spectroscopy (ATR-FTIR)

**Figure 4:**
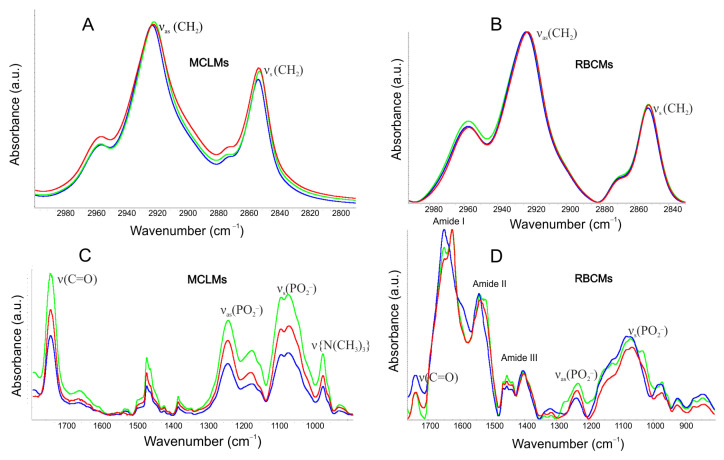
FTIR-ATR spectra in the range of 3000–2840 cm^−1^ (**A**,**B**) and 1750–900 cm^−1^ (**C**,**D**) regions for MCLMs (**A**,**C**) and RBCMs (**B**,**D**) measured at 37 °C. Red curves—control (RBCMs or MCLMs), blue curves—control + DMSO, and green curves—membrane modified with iodolactone **LV2** (50 µM).

ATR-FTIR spectroscopy was employed as a highly sensitive tool to elucidate the molecular-level interactions between the iodolactone **LV2** and the constituents of the erythrocyte (RBCMs) and cancer-mimicking (MCLMs) membranes. Specifically, perturbations were investigated in the hydrophilic headgroups, the hydrophobic core (via methylene chain packing and fluidity) and the bilayer interface (represented by ester groups). In RBCMs, ATR-FTIR provided insights into membrane protein conformational stability through the Amide I and II vibrational modes. Figure 4B,D presents comparative infrared spectra for the control (RBCMs treated with DMSO) and the experimental (RBCMs treated with iodolactone **LV2**) groups, highlighting site-specific effects on membrane structure.

Within lipid systems, the most prominent signals correspond to CH_2_ stretching vibrations, typically appearing in the 3000–2800 cm^−1^ range. An upward shift in these wavenumbers reflects enhanced fluidity within the membrane’s hydrophobic core. Notably, iodolactone **LV2** did not alter the frequency of the hydrocarbon chain signals, suggesting that the compound does not substantially perturb the hydrophobic regions of the lipid bilayer (Figure 4A,B).

To evaluate the interaction between iodolactone **LV2** and the polar head groups of MCLMs, the symmetric and asymmetric phosphate stretching bands, along with choline vibrations, were analyzed (Figure 4C). The asymmetric stretching vibration of PO_2_^−^ groups (ν_as_(PO_2_^−^)) occurs between 1220 and 1260 cm^−1^, and is highly sensitive to environmental polarity and hydrogen bonding. Typically, increased hydration shifts this band to lower wavenumbers. This effect was observed in the studied system, as iodolactone **LV2** induced a slight shift to lower values (from 1237.95 cm^−1^ in MCLMs to 1236.6 cm^−1^ in the presence of **LV2**). A corresponding shift occurred in the symmetric vibration (ν_s_(PO_2_^−^)), moving from 1087.71 cm^−1^ to 1086.22 cm^−1^. These wavenumber alterations suggest that iodolactone **LV2** induces subtle conformational changes in the polar lipid regions.

In the polar region of the lipid spectra, a band corresponding to choline vibrations was identified at ν_as_(N-C) = 969.3 cm^−1^ for the control sample (MCLMs + DMSO). Spectral analysis revealed that iodolactone **LV2** did not induce significant shifts in this range. A similar lack of substantial changes was observed in the ester group region (1750–1700 cm^−1^) (Figure 4C,D). In contrast, discrete spectral changes appeared in the Amide I and Amide II band regions for RBCMs (Figure 4D). These modifications primarily involved changes in band intensity rather than significant shifts in peak positions, suggesting that membrane proteins may undergo alterations in their local environment or minor conformational rearrangements upon interaction with iodolactone **LV2**.

Since the Amide I band originates primarily from the C=O stretching vibrations of peptide bonds, changes in this band suggest alterations in the local protein environment and possible conformational changes. The absence of analogous changes in the lipid bands of RBCMs (both hydrophobic and polar) suggests that the primary binding sites for iodolactone **LV2** in this system may be associated with protein domains rather than the lipid matrix.

## 3. Materials and Methods

### 3.1. Free-Radical Scavenging Assays

The antiradical capacity of the tested compounds was evaluated using the DPPH^•^ radical and the ABTS^•+^ cation radical. The DPPH assay was conducted according to the method described by Brand-Williams et al. [78], while the ABTS assay followed the procedure reported by Re et al. [79] with minor modifications in both cases.

Halolactones were initially dissolved in biological-grade DMSO to prepare stock solutions and subsequently diluted to working concentrations of 6.25, 12.5, 25, 50, 100, 200, 300, 400, 500, 600, 800, 1000, 1500, 2000, and 2500 µM. The same procedure was applied for Trolox, used as the positive control, which was tested at concentrations of 5, 15, 25, 35, and 50 μM (in ABTS assay) and 25, 50, 75, and 100 μM (in DPPH assay). Each assay was performed in a total volume of 200 μL, containing either DPPH^•^ radical solution in methanol (54 μM) or ABTS^•+^ radical solution in water (25 μM) along with the test compound solution. Control samples for both assays were prepared without the test compound. The final concentration of DMSO in all samples did not exceed 1% (*v*/*v*). All experiments were conducted in triplicate. The tests were conducted in 96-well microplates (Greiner BIO-ONE, Kremsmünster, Austria) and incubated at 25 °C in the dark for 15 min with continuous gentle mixing. Following incubation, absorbance was measured using a microplate spectrophotometer (EPOCH, BioTek, Santa Clara, CA, USA) at 517 nm (DPPH^•^) and 734 nm (ABTS^•+^).

The degree of radical scavenging was calculated based on the decrease in absorbance of the radical solution relative to the control sample, using the following formula:

$$Radical\;scavenging\;activity\;[\%]=\frac{A_{control}-A_{sample}}{A_{control}}\times 100\%$$

where *A_control_* is the absorbance of the DPPH^•^ or ABTS^•+^ solution without the tested compound and *A_sample_* is the absorbance of the corresponding radical solution in the presence of the tested compound.

Results are presented as EC_50_ values, defined as the concentration required to achieve 50% of the maximal scavenging activity against the DPPH^•^ or ABTS^•+^ radicals, respectively. Statistical analysis was performed separately for each radical.

### 3.2. Thiobarbituric Acid Reactive Substances Assay (TBARS Assay)

#### 3.2.1. Preparation of Phosphatidylcholine Model Lipid Membranes (PLMs)

PLMs were prepared according to the method of Strugała et al. [80], with minor modifications. Model lipid membranes composed of phosphatidylcholine from egg yolk (EPC) were prepared by dissolving the lipid in chloroform, followed by evaporation under a nitrogen stream to form a thin lipid film. The film was then dried under vacuum in a desiccator for 1 h to remove residual solvent. The lipid film was hydrated with phosphate buffer (PBS, pH 7.4) and vortexed thoroughly. The resulting suspension was sonicated for 15 min in a cooled water bath (0 °C) using a 20 kHz sonicator (Sonic, Milan, Italy).

#### 3.2.2. TBARS Assay

The TBARS assay was conducted according to the method described by Włoch et al. [81], with minor modifications in both cases.

Test compounds were initially dissolved in biological-grade DMSO to prepare stock solutions and subsequently diluted to working concentrations of 25, 50, 100, 200, 400, and 800 μM (in the case of halolactones) and 10, 20, 40, 60, and 80 μM (in the case of Trolox used as the positive control) with the final DMSO concentration in all samples not exceeding 1% (*v*/*v*). Control samples were prepared without the test compound. All experiments were conducted in triplicate.

Lipid peroxidation was induced by UV-C radiation (λ = 200–280 nm) at an intensity of 3.5 mW·cm^−2^ using a bactericidal lamp (UVP radiometer, Analytik Jena AG, Jena, Germany). Samples were exposed to UV-C radiation for 60 min.

The degree of lipid peroxidation was quantified as the concentration of malondialdehyde (MDA) generated during the process. MDA forms a colored complex with thiobarbituric acid (TBA), which was measured spectrophotometrically at 535 nm using a microplate spectrophotometer (EPOCH, BioTek, Santa Clara, CA, USA).

Antioxidant activity was expressed as the percentage inhibition of lipid peroxidation relative to the control sample (without test compounds), calculated after 60 min of exposure using the following formula:

$$Inhibition\;[\%]=\frac{A_{UVC}-A_{U}}{A_{UVC}}\times 100\%$$

where *A_UVC_* is the absorbance of the control samples and *A_U_* is the absorbance of samples with test compounds.

For comparative purposes, IC_50_ values were determined, representing the concentration required to inhibit 50% of the lipid peroxidation.

### 3.3. Anti-Inflammatory Activity

The anti-inflammatory potential of the tested compounds was evaluated using the cyclooxygenase enzymes COX-1 and COX-2. Enzyme inhibition was measured with a commercial COX Colorimetric Inhibitor Screening Assay Kit (Cayman Chemical, Ann Arbor, MI, USA) according to the manufacturer’s instructions.

In brief, the assay was performed in 96-well microplates (Greiner BIO-ONE, Kremsmünster, Austria). Test compounds were initially dissolved in biological-grade DMSO to prepare stock solutions and subsequently diluted to working concentrations of 50, 100, 200, 300, 600, and 800 μM in the case of halolactones and 25, 50, 100, and 300 μM in the case of ibuprofen used as a positive control. The final DMSO concentration in all samples did not exceed 1% (*v*/*v*). Each 180 µL sample contained assay buffer (0.1 M Tris-HCl, pH 8.0), the heme solution (102.6 µM), and diluted COX-1 or COX-2 enzyme preparation (1 mg/mL) along with the test compound solution. Control samples were prepared without the test compound. Each compound was tested in three independent experiments.

The tested solutions were incubated for 5 min at room temperature in the dark with gentle mixing. A colorimetric substrate solution (24.35 mM) was then added, immediately followed by an arachidonic acid solution (35 mM), bringing the final sample volume to 220 µL. Microplates were incubated for an additional 2 min at room temperature in the dark.

Absorbance changes were measured at 590 nm using a microplate spectrophotometer (EPOCH, BioTek, Santa Clara, CA, USA). IC_50_ values were determined from percentage inhibition data using the following formula:

$$Inhibition\;[\%]=\frac{A_{COX}-A_{C}}{A_{COX}}\times 100\%$$

where *A_COX_* is the absorbance of the control samples (COX-1 or COX-2) and *A_C_* is the absorbance of samples with test compounds.

Statistical analysis was performed separately for each enzyme.

### 3.4. Antiproliferative Activity

#### 3.4.1. Cell Lines and Cell Culture Conditions

The cell lines A549 (human lung adenocarcinoma), MCF-7 (breast cancer), LM-MEL-75 (human melanoma), HeLa (cervical cancer), and Saos-2 (osteosarcoma) were purchased from the American Type Culture Collection (Rockville, MD, USA). The cell lines A2780 (human ovarian carcinoma) and EPG85-257RDB (human gastric adenocarcinoma) were kindly provided by Associate Professor T. Gębarowski, Division of Animal Anatomy, Department of Biostructure and Animal Physiology, Wrocław University of Environmental and Life Sciences, Poland. Normal human dermal fibroblasts (NHDFs), used as a normal cell line, were purchased from PromoCell (Heidelberg, Germany).

NHDF cells were cultured in DMEM supplemented with 10% FBS, whereas all neoplastic cell lines (A549, LM-MCF-7, LM-MEL-75, HeLa, Saos-2, A2780, and EPG85-257RDB) were maintained in RPMI-1640 medium supplemented with 10% FBS, L-glutamine (4 nM), streptomycin (100 U/mL), and penicillin (100 μg/mL).

All cell cultures were maintained in 75 cm^2^ culture flasks (Eppendorf, Hamburg, Germany) under humidified conditions at 37 °C with 5% CO_2_ in a SafeGrow Pro incubator (EuroClone, Pero, Italy). Cells were detached from the bottom of the culture flask using a solution of 0.25% trypsin and 0.02% EDTA in Hanks′ Balanced Salt Solution with phenol red and passaged every 5–7 days.

#### 3.4.2. MTT Assay

This quantitative assay was performed following Annex C of ISO 10993-5 [82]. The tested cells were seeded in 96-well culture plates (Eppendorf, Hamburg, Germany) at a density of 3 × 10^3^ cells per well in 100 µL of the appropriate growth medium and incubated for 24 h to allow for cell attachment prior to treatment.

Stock solutions were prepared for each experiment by dissolving 3.32 mg of the tested compound in 1 mL of biological-grade DMSO. Subsequent dilutions of stock solutions with culture medium yielded final working concentrations in the range of 0.5–20 µg/mL (0.625, 1.25, 2.5, 5, 10, and 20 µg/mL). The final DMSO concentration in each well was maintained below 1%, a level generally considered non-toxic to cells [83].

After 24 h of treatment, an MTT solution (1 mg/mL in growth medium) was added to all wells. Samples were incubated under the same conditions for 2 h. Following incubation, the supernatant was carefully removed and replaced with 100 µL of isopropanol.

Absorbance was measured at 570 nm (reference 630 nm) using a Multiskan GO microplate spectrophotometer (Thermo Fisher Scientific, Waltham, MA, USA) to correct for background absorbance and reduce measurement artifacts, as formazan shows negligible absorbance at 630 nm.

Untreated cells served as the negative control, while doxorubicin was tested as the positive control at concentrations of 0.5, 1.0, 2.0, and 4.0 μg/mL. The IC_50_ values for the tested compounds were calculated as the concentration causing 50% inhibition of cell proliferation. All experiments comprised four independent biological replicates, each performed in quadruplicate, with the results presented as mean IC_50_ ± SD. Statistical analysis was performed separately for each cell line.

### 3.5. Shape of Red Blood Cells (RBCs)

Human erythrocytes were obtained from a Blood Donation Center in Wrocław and prepared according to the protocol described in the Materials and Methods (see Section 3.6.1). After washing, the hematocrit value was determined and the erythrocyte suspension was adjusted with physiological sodium chloride solution (0.9% NaCl) to a final hematocrit of 12%.

Iodolactone **LV2** was dissolved in biological-grade DMSO. Stock solutions were diluted to working concentrations of 5, 25, and 50 μM. Control samples were prepared without the test compound, while vehicle controls contained DMSO at equivalent concentrations. The DMSO concentration in all samples did not exceed 1% (*v*/*v*). Each test sample had a total volume of 1 mL, consisting of 897.5 μL of saline solution (0.9% NaCl), 100 μL of erythrocytes, and the test compound solution. The final erythrocyte concentration in all samples was 1.2% hematocrit.

Samples were incubated at 37 °C under static conditions for 1 h. After incubation with the test compound, erythrocytes were fixed with 2.5% glutaraldehyde for 30 min.

Erythrocyte morphology was examined using a Nikon ECLIPSE E200 optical microscope (Nikon Europe B.V., Amstelveen, the Netherlands) equipped with a MOTI CAM S6 camera (Motic Europe, S.L.U., Barcelona, Spain) at 100× magnification with an oil immersion objective (Fluka, Sigma-Aldrich^®^, Steinheim, Germany).

Erythrocyte shapes were classified according to the Bessis and Brecher scale [84], which assigns morphological indices to three basic forms: discocytes (D), stomatocytes (S), and echinocytes (E). Erythrocyte morphology was evaluated by visual inspection under a microscope and individual cell types were counted in each microscopic field of view. The results were expressed as a percentage of each morphological type relative to the total number of analyzed cells. Intermediate forms representing transitional stages between these basic morphologies (e.g., discostomatocytes, discoechinocytes, spheroechinocytes and spherostomatocytes) were also identified and quantified based on established morphological criteria described in the literature [84]. Classification was performed according to characteristic shape features, including gradual changes in membrane contour, the degree of echinocytic or stomatocytic transformation, and overall cell sphericity, thereby allowing the assignment of cells to intermediate categories. All experiments were performed in triplicate. Statistical analysis was performed separately for each erythrocyte morphological form.

### 3.6. Fluorometric Methods

The effects of the newly synthesized compound on membrane fluidity and lipid packing were investigated using three types of model membranes: those formed from lipids isolated from erythrocytes (LEMs), erythrocyte membranes (RBCMs), and those mimicking cancer cell lipid bilayers (MCLMs).

#### 3.6.1. Preparation of Red Blood Cells (RBCs)

Human red blood cells (erythrocytes) were isolated from whole blood collected from healthy donors at the Blood Donation Center in Wrocław. According to Polish regulations, the use of human erythrocytes from blood donation centers for research purposes does not require ethics committee approval.

Fresh blood (0.5 L) was centrifuged at 2500 rpm for 3 min at 4 °C and the erythrocytes were subsequently washed three times with physiological saline (0.9% NaCl) to remove residual plasma and contaminants.

#### 3.6.2. Preparation of Red Blood Cell Membranes (RBCMs)

The isolation of RBCMs followed the method of Dodge et al. [85] with minor modifications according to Włoch et al. [86].

Isolated red blood cells (RBCs, see Section 3.6.1) were washed four times with 310 mOsm isotonic phosphate-buffered saline (PBS) by centrifugation at 2500 rpm for 3 min at 4 °C, discarding the supernatant after each step. The erythrocytes were then resuspended in 310 mOsm PBS to a hematocrit of 60%.

For hemolysis, hypotonic 20 mOsm PBS was added dropwise (1:14 *v*/*v*) to the gently stirred suspension. The hemolysate was incubated at 4 °C for 1 h to promote osmotic swelling and hemoglobin release, then centrifuged at 12,000 rpm for 15 min at 4 °C; the hemoglobin-containing supernatant was discarded.

The membrane pellet was washed three times with cold 20 mOsm PBS under the same centrifugation conditions until a creamy white or slightly pink pellet was obtained, confirming hemoglobin removal. The protein content of the resulting RBCMs was quantified using the Lowry method with the Folin–Ciocalteu reagent [87].

#### 3.6.3. Preparation of Lipids from Erythrocyte Membranes (LEMs)

Natural lipids were extracted from erythrocyte membranes (RBCMs, see Section 3.6.2) following the method described by Maddy [88], with minor modifications.

An RBCM suspension (10 mL) was cooled to 0 °C in an ice bath. Ice-cold *n*-butanol (7.5 mL) was added and the sample was vigorously mixed in the ice bath for 20 min. The mixture was centrifuged at 14,000 rpm for 5 min, separating into an *n*-butanol phase (containing lipids), an aqueous phase (containing proteins), and a thin interfacial layer of insoluble protein. The *n*-butanol phase was carefully collected and the solvent was evaporated to dryness to yield a lipid residue. The lipid pellet was dissolved in a chloroform:methanol mixture (1:1, *v*/*v*). Total lipid content was determined by triple extraction of a known weight of freeze-dried material using the same solvent system.

To prepare lipid membranes, the lipid solution in chloroform:methanol (1:1, *v*/*v*) was evaporated under a nitrogen stream and dried in a vacuum desiccator for 2 h to form a thin lipid film and ensure the complete removal of residual solvent. The lipid film was hydrated with phosphate buffer (pH 7.4) and thoroughly vortexed for uniform hydration. The resulting suspension was sonicated for 15 min in a cooled water bath (0 °C) using a 20 kHz sonicator (Sonic, Milan, Italy) to yield a homogeneous lipid dispersion.

#### 3.6.4. Preparation of Mimic Cancer Lipid Membrane Model (MCLMs)

The lipid composition of the MCLMs was as follows: POPC (34.0 mol%), POPE (23.1 mol%), SM (11.0 mol%), PI (7.8 mol%), SOPS (8.0 mol%), and cholesterol (16.1 mol%). All lipids were initially dissolved in chloroform following the method described by Strugała et al. [89], with minor modifications.

The lipid mixture was evaporated under a nitrogen stream and dried in a vacuum desiccator for 2 h to form a thin lipid film and remove residual solvent. The film was hydrated with phosphate buffer (pH 7.4) to a final lipid concentration of 0.1 mg/mL and vortexed to produce a homogeneous, milky suspension. The suspension was sonicated for 10 min at 20 kHz and 80% amplitude in a cooled water bath (0 °C) using a sonicator (Sonic, Milan, Italy).

#### 3.6.5. General Procedure for Fluorometric Methods

The effect of iodolactone **LV2** on the fluidity and packing of lipids from erythrocytes (LEMs), red blood cell membranes (RBCMs), and model cancer cell lipid membranes (MCLMs) was investigated using a fluorimetric method previously described by Włoch et al. [90], with minor modifications. Four fluorescent probes: MC540, Laurdan, DPH and TMA-DPH, which localize in different membrane regions, were employed to assess these properties.

For the assays, model membranes with fluorescent probes were prepared, containing either LEMs (0.1 mg/mL), RBCMs (protein content 6.2 µg/mL), or MCLMs (0.1 mg/mL) suspended in isotonic phosphate buffer (PBS, pH 7.4) and combined with fluorescent probes (1 µM). The mixture was incubated for 30 min at room temperature in the dark with gentle mixing. The mixture was then combined with a DMSO solution of **LV2** to achieve final concentrations of 5–50 µM (5, 10, 20, 30, 40, and 50 µM), yielding a total assay volume of 1 mL. The final DMSO content in all samples did not exceed 1% (*v*/*v*).

Prepared samples were incubated at 37 °C for 1 h in the dark with gentle mixing. Fluorescence measurements were performed at 37 °C using a Cary Eclipse fluorimeter (Varian, San Diego, CA, USA). Two separate control samples were prepared: a probe control without DMSO and a probe control with DMSO at the same final concentration as in the corresponding test samples. Three independent experiments were performed for each compound.

Excitation and emission wavelengths for the DPH and TMA-DPH probes were λ_ex_ = 360 nm, λ_em_ = 425 nm and λ_ex_ = 360 nm, λ_em_ = 428 nm, respectively. Laurdan fluorescence was excited at λ_n_ = 360 nm, with emission recorded at λ_n_ = 440 and λ_n_ = 490 nm. Merocyanine 540 fluorescence intensities were measured from λ_em_ = 560 nm to λ_em_ = 650 nm, with maximum intensity at λ = 584 nm.

Fluorescence anisotropy (A) of the DPH and TMA-DPH probes was calculated using the formula described by Lakowicz et al. [91]:

$$A=\frac{I_{II}-{GI}_{⊥}}{{(I}_{II}+{2GI}_{⊥})},$$

where $I_{II}$ and $I_{⊥}$ represent the fluorescence intensities observed parallel and perpendicular, respectively, to the polarization direction of the exciting light and *G* is the instrument correction factor, dependent on emission wavelength.

The generalized polarization (GP) parameter for Laurdan was calculated according to Parasassi et al. [71]:

$$G=\frac{I_{b}-I_{r}}{{(I}_{b}+I_{r})},$$

where $I_{b}$ and $I_{r}$ correspond to fluorescence intensities at emission wavelengths of λ_em_ = 440 nm and λ_em_ = 490 nm.

Statistical comparisons were performed separately for each membrane type.

### 3.7. Attenuated Total Reflectance-Fourier Transform Infrared Spectroscopy (ATR-FTIR)

The ATR-FTIR methodology was based on the method reported by Pruchnik et al. [92], with minor modifications.

Iodolactone **LV2** was dissolved in DMSO to achieve a final concentration of 50 µM. Measurements were performed on RBCMs and MCLMs. The RBCM suspension in phosphate buffer (PBS, pH 7.4) and the MCLMs in phosphate buffer or Milli-Q water were supplemented with the test compound solution or DMSO (control) at the same final concentration as in the corresponding test samples. Samples with the compound were incubated for 1 h at 37 °C. The suspensions were then applied to ZnSe ATR crystal plates (Thermo Fisher Scientific, Waltham, MA, USA) and dehydrated. Spectral measurements were conducted at 37 °C using a thermostat. At each measurement point, spectra were collected in triplicate, with individual spectra generated by averaging 128 scans at 2 cm^−1^ resolution over the 700–4000 cm^−1^ range. Background spectra were recorded for the pure ZnSe crystal in PBS (pH 7.4) and Milli-Q water, as experimental controls.

All experiments were conducted using a Nicolet 6700 FTIR spectrometer (Thermo Fisher Scientific, Waltham, MA, USA) and data were analyzed using OMNIC 8.3.103 software (Thermo Nicolet, Thermo Fisher Scientific, Waltham, MA, USA). Measurements were replicated on two independent ATR plates across three independent experiments to ensure reproducibility.

### 3.8. Statistical Analysis

Analyses were performed using GraphPad Prism 8 software (GraphPad Software, San Diego, CA, USA). Prior to hypothesis testing, data distribution normality was assessed. Differences between two groups were evaluated using Student’s *t*-test, whereas comparisons among more than two groups were analyzed using one-way analysis of variance (ANOVA). Depending on the experimental design, post hoc analyses were performed using Dunnett’s multiple comparison test for comparisons with the control group or Tukey’s multiple comparison test for pairwise comparisons among treated groups.

For analyses performed using Tukey’s test, values not sharing a common superscript letter (a, b, c, etc.) were considered significantly different (*p* < 0.05). For analyses performed using Dunnett’s test, statistical significance relative to the control group was denoted as follows: ns—not significant, * *p* < 0.05, ** *p* < 0.01, *** *p* < 0.001, and **** *p* < 0.0001.

## 4. Conclusions

In this study, a series of novel racemic vanillin-derived γ-halo-δ-lactones and δ-halo-γ-lactones bearing a phenolic ring at the β-position were evaluated for their antioxidant activity and lipid-protective effects. All compounds exhibited significantly higher antioxidant activity than the starting compound—vanillin (**V**). The iodolactone *trans*-4-(4′-hydroxy-3′-methoxyphenyl)-5-(1-iodoethyl)dihydrofuran-2(3H)-one (**LV2**) showed the highest antioxidant activity in the ABTS free-radical scavenging assay and was the most effective at inhibiting lipid peroxidation (TBARS), comparable to the reference antioxidant Trolox^®^. The antioxidant and lipid peroxidation assays further confirmed a clear structure–activity relationship, indicating that the *trans* configuration of the substituents at the γ-lactone ring and the presence of iodine strongly enhance the biological activity of vanillin-derived δ-halo-γ-lactones. Iodolactone **LV2** was also the only compound to demonstrate measurable anti-inflammatory activity among all tested halolactones. In broader antiproliferative studies, iodolactone **LV2** displayed significant cytotoxicity against human melanoma (LM-MEL-75), gastric adenocarcinoma (EPG85-257RDB), and ovarian carcinoma (A2780) cells while maintaining low toxicity against normal NHDFs. This multifunctional bioactivity, together with our previous studies—showing no toxicity against human erythrocytes and antiproliferative activity against canine and human cancer cell lines without affecting normal NIH/3T3 fibroblasts—supports the potential of iodolactone **LV2** as a promising candidate for further investigations into the mechanisms underlying its anticancer activity. To elucidate its interactions with biological membranes, we investigated the effects of iodolactone **LV2** on membranes formed from lipid extract-based membranes (LEMs), intact erythrocyte membranes (RBCMs), and a mimic of cancer cell lipid bilayers (MCLMs). Morphological changes in erythrocyte shapes as well as spectroscopic methods revealed that iodolactone **LV2** primarily interacts with polar membrane regions and membrane proteins, with minimal disturbance of the hydrophobic core, indicating a selective mode of action. Together, these analyses provide a comprehensive overview of this compound as a multifunctional bioactive agent.

Future studies will focus on the synthesis of optically pure enantiomers of iodolactone **LV2** to compare their activities. Additionally, we aim to elucidate the mechanisms underlying its antiproliferative effects, including detailed investigations of cell cycle regulation and apoptosis induction in cancer cells. Finally, we will explore the development of phospholipid-based liposomal nanocarriers containing iodolactone **LV2** to improve targeted delivery and enhance its therapeutic efficacy against cancer cells.

## Figures and Tables

**Table 1:** Antioxidant activity of the tested vanillin (**V**) and vanillin-derived lactones (**LV1**–**LV5**) expressed as EC_50_ values (µM) in the scavenging of DPPH^•^ and ABTS^•+^ free radicals.

Compound	Type of Free Radical	
	DPPH^•^	ABTS^•+^
	EC_50_ [µM] ^1^	
**V**	no activity	938.94 ± 28.53 ^a^
**LV1**	810.53 ± 11.51 ^b^	125.88 ± 21.60 ^c^
**LV2**	293.82 ± 7.81 ^cd^	35.67 ± 2.34 ^d^
**LV3**	663.50 ± 3.80 ^bc^	80.44 ± 8.95 ^cd^
**LV4**	661.17 ± 41.14 ^bc^	129.95 ± 46.49 ^c^
**LV5**	2217.73 ± 413.49 ^a^	401.5 ± 38.44 ^b^
Trolox^®^	51.96 ± 2.88 ^d^	31.42 ± 1.29 ^d^

^1^ The data represent mean values  ±  SD (n = 3). Statistical analysis was performed using one-way analysis of variance (ANOVA), followed by Tukey’s multiple comparison test for pairwise comparisons among groups. Values not sharing a common superscript letter (a, b, c, d, etc.) within the same column were considered significantly different at *p* < 0.05.

**Table 2:** Inhibition of UV-C-induced oxidation by vanillin (**V**) and vanillin-derived lactones (**LV1**–**LV5**).

Compound	IC_50_ [µM] ^1^
**V**	no activity
**LV1**	97.21 ± 19.50 ^cd^
**LV2**	48.67 ± 5.46 ^d^
**LV3**	146.7 ± 14.50 ^c^
**LV4**	545.98 ± 14.96 ^b^
**LV5**	663.27 ± 65.01 ^a^
Trolox^®^	64.56 ± 9.60 ^d^

^1^ The data represent mean values  ±  SD (n = 3). Statistical analysis was performed using one-way analysis of variance (ANOVA), followed by Tukey’s multiple comparison test for pairwise comparisons among groups. Values not sharing a common superscript letter (a, b, c, d, etc.) within the same column were considered significantly different at *p* < 0.05.

**Table 3:** Inhibition of COX-1 and COX-2 enzymatic activity expressed as IC_50_ (µM) values for the tested compounds.

Compound	COX-1 ^1^	COX-2
**LV2**	294.21 ± 50.54	608.90 ± 4.93
Ibuprofen	205.70 ± 39.05	95.30 ± 4.07

^1^ The data represent mean values  ±  SD (n = 3).

**Table 4:** Antiproliferative activity of vanillin (**V**) and the iodolactone **LV2** against a panel of cell lines after 24 h of treatment, expressed as IC_50_ values (µM).

Compound	Cell Line							
	A549	LM-MEL-75	MCF-7	HeLa	EPG85-257RDB	A2780	Saos-2	NHDF
	IC_50_ [µM] ^1^							
**V**	24.52 ± 10.45 ^a^	36.28 ± 17.15 ^a^	>50.00 ^a^	>50.00 ^a^	33.65 ± 6.38 ^a^	>50.00 ^a^	>50.00 ^a^	>50.00 ^a^
**LV2**	33.92 ± 2.35 ^a^	8.67 ± 9.82 ^b^	>50.00 ^a^	>50.00 ^a^	7.37 ± 3.22 ^b^	13.62 ± 7.38 ^b^	37.90 ± 5.42 ^b^	>50.00 ^a^
Doxorubicin	0.68 ± 0.24 ^b^	2.19 ± 1.38 ^b^	1.32 ± 0.53 ^b^	1.03 ± 0.42 ^b^	5.48 ± 0.75 ^b^	1.31 ± 1.17 ^b^	0.88 ± 0.68 ^c^	0.44 ± 0.31 ^b^

^1^ The data represent mean values ± SD (n = 4), each measurement was performed in quadruplicate (four wells per experiment). Statistical analysis was performed using one-way analysis of variance (ANOVA), followed by Tukey’s multiple comparison test for pairwise comparisons among groups. Values not sharing a common superscript letter (a, b, c) within the same column were considered significantly different at *p* < 0.05.

**Table 5:** Selectivity index (SI) of vanillin (**V**) and the vanillin-derived iodolactone **LV2** against selected cancer cell lines.

Compound	Cell Line				
	A549	LM-MEL-75	EPG85-257RDB	A2780	Saos-2
**V**	>2.04 ^1^	>1.37	>1.49	-	-
**LV2**	>1.47	>5.77	>6.78	>3.67	>1.32
Doxorubicin	0.65	0.20	0.08	0.34	0.50

^1^ SI calculated as the ratio of the IC_50_ value obtained for the normal cell line (NHDFs) to that measured for the corresponding cancer cell line.

**Table 6:** Percentage change in MC540 probe fluorescence intensity in LEMs, RBCMs, and MCLMs at λ = 584 nm in the presence of iodolactone **LV2**.

Concentration [µM]	Model Membrane		
	LEMs	RBCMs	MCLMs
Control + DMSO	↑ 5.4 ^1^	↑ 24.1	↓ 2.0
5	↑ 7.5	↑ 42.3	↑ 3.5
10	↑ 20.8	↑ 37.4	↑ 8.0
20	↑ 18.3	↑ 56.0	↑ 7.7
30	↑ 23.1	↑ 77.8	↑ 9.7
40	↑ 23.0	↑ 51.3	↑ 7.8
50	↑ 13.0	↑ 70.3	↑ 7.9

^1^ The data represent mean values (n = 3). ↑ and ↓ indicate an increase or decrease in fluorescence intensity relative to the control.

## Data Availability

The datasets used and/or analyzed during the current study are available from the corresponding author upon reasonable request.

## Supplementary Materials

The following supporting information can be downloaded at: https://www.mdpi.com/article/10.3390/ijms27114821/s1.
