# Supplementary material for: Multifunctional Bioactivity of Halolactones Derived from Vanillin and Their Effects on Lipid Membranes: Biological and Biophysical Evaluation
Source: Int J Mol Sci. 2026 May 27;27(11):4821. doi: 10.3390/ijms27114821 (PMC13256543; doi:10.3390/ijms27114821)
Supplement: Supplementary file 1 [file ijms-27-04821-s001.zip › ijms-4295241-supplementary.pdf]

Supplementary Information

Multifunctional Bioactivity of Halolactones Derived from Vanillin and Their Effects on Lipid Membranes: Biological and Biophysical Evaluation

Anna Dunal[1,*], Aleksandra Włoch[2], Dominik Poradowski[3], Aleksander Chrószcz[3], Witold Gładkowski[1] and Hanna Pruchnik[2]

[1] Department of Food Chemistry and Biocatalysis, Faculty of Biotechnology and Food Sciences, Wrocław University of Environmental and Life Sciences, Norwida 25, 50-375 Wrocław, Poland; witold.gladkowski@upwr.edu.pl (W.G);

[2] Department of Physics and Biophysics, Faculty of Biotechnology and Food Sciences, Wrocław University of Environmental and Life Sciences, Norwida 25, 50-375 Wrocław, Poland; aleksandra.wloch@upwr.edu.pl (A.W.); hanna.pruchnik@upwr.edu.pl (H.P.);

[3] Department of Biostructure and Animal Physiology, Division of Animal Anatomy, Faculty of Veterinary Medicine, Wroclaw University of Environmental and Life Sciences, Kożuchowska 1, 51-631 Wrocław, Poland; dominik.poradowski@upwr.edu.pl (D.P); aleksander.chroszcz@upwr.edu.pl (A.C.);

* Correspondence: anna.dunal@upwr.edu.pl (A.D.)

Table of content

	Pages
Scheme S1. Synthesis of vanillin-derived halolactones.	3
Figure S1. Percentage distribution of erythrocyte morphological forms induced by iodolactone LV2: (A) spherostomatocytes (SST(-4)), (B) second-order stomatocytes (ST2(-3)), (C) first-order stomatocytes (ST1(-2)), (D) discostomatocytes (DST(-1)), (E) discocytes (D(0)), (F) discoechinocytes (DE(1)), (G) echinocytes (E(2)), (H) spheroechinocytes (SE(3)) and (I) spherocytes (S(4)).	3-4
Figure S2. Concentration-dependent changes in MC540 fluorescence intensity in (A) LEMs, (B) RBCMs and (C) MCLMs following treatment with iodolactone LV2.	5
Figure S3. Changes in Laurdan generalized polarization (GP) in (A) LEMs, (B) RBCMs and (C) MCLMs following treatment with iodolactone LV2.	5
Figure S4. Changes in DPH anisotropy (A) in (A) LEMs, (B) RBCMs and (C) MCLMs following treatment with iodolactone LV2.	6
Figure S5. Changes in TMA-DPH anisotropy (A) in (A) LEMs, (B) RBCMs and (C) MCLMs following treatment with iodolactone LV2.	6
1. Chemicals and Reagents	7
1.1. Free-Radical Scavenging Assays	7
1.2. Chemicals for TBARS Assay	7
1.3. Chemicals for MTT Tests	7
1.4. Chemicals for Fluorometric Methods	7

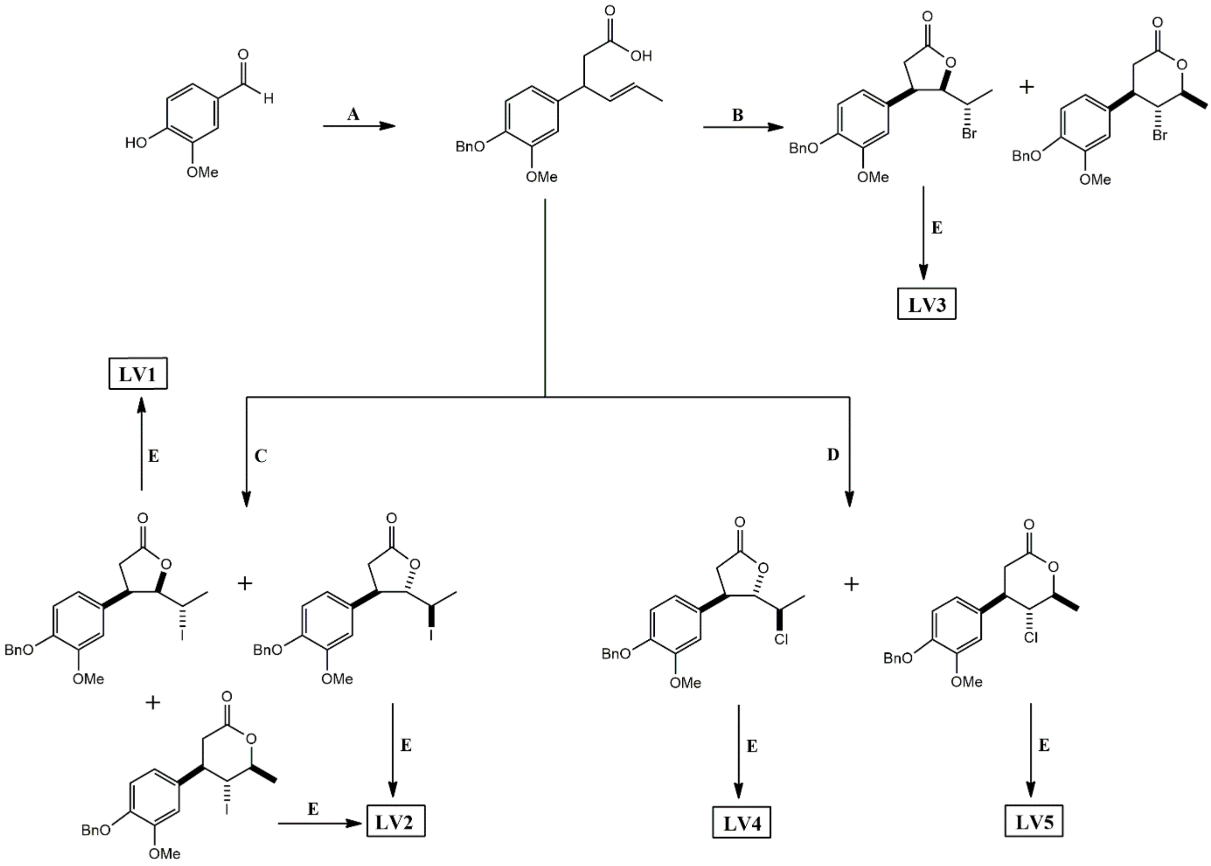

Scheme S1. Synthesis of vanillin-derived halolactones. *Reagents and conditions:* (**A**) (**I**) BnBr, K_2CO_3, EtOH, r.t., 48 h; (**II**) acetone, 10% NaOH, r.t., 24 h; (**III**) MeOH, $NaBH_4$, ice bath 1 h, then r.t., 23 h; (**IV**) $MeC(OEt)_3$, EtCOOH, 138°C, 6 h; (**V**) 10% NaOH, EtOH, reflux, 5 h; (**B**) NBS, acetic acid, THF, r.t., 24 h; (**C**) I_2/KI, Et_2O, $NaHCO_3$, r.t., 20 h; (**D**) NCS, acetic acid, THF, r.t., 24 h; (**E**) $FeCl_3$, CH_2Cl_2, r.t., 0.5 h.

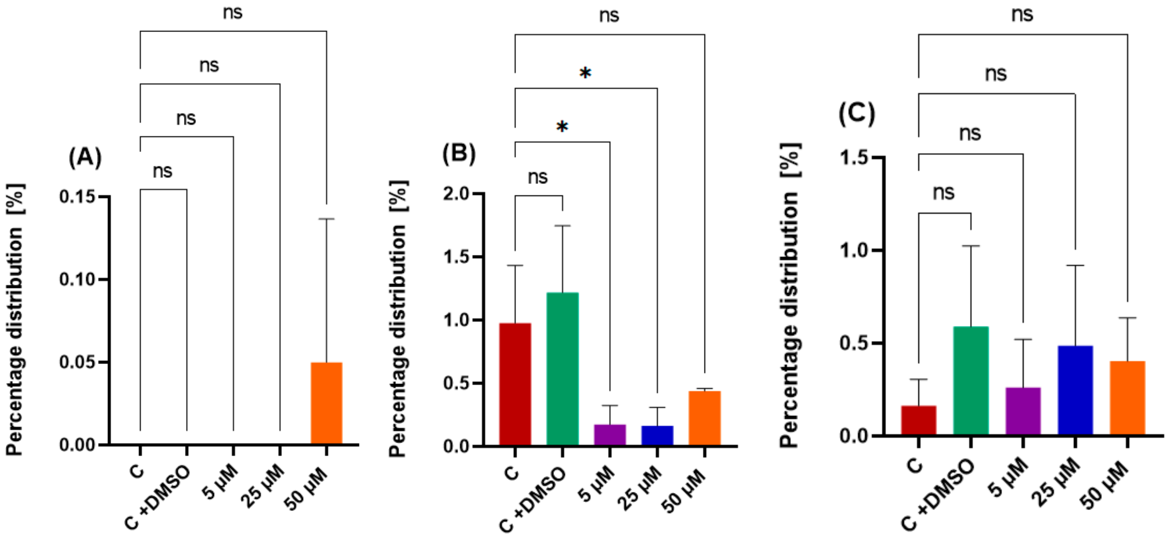

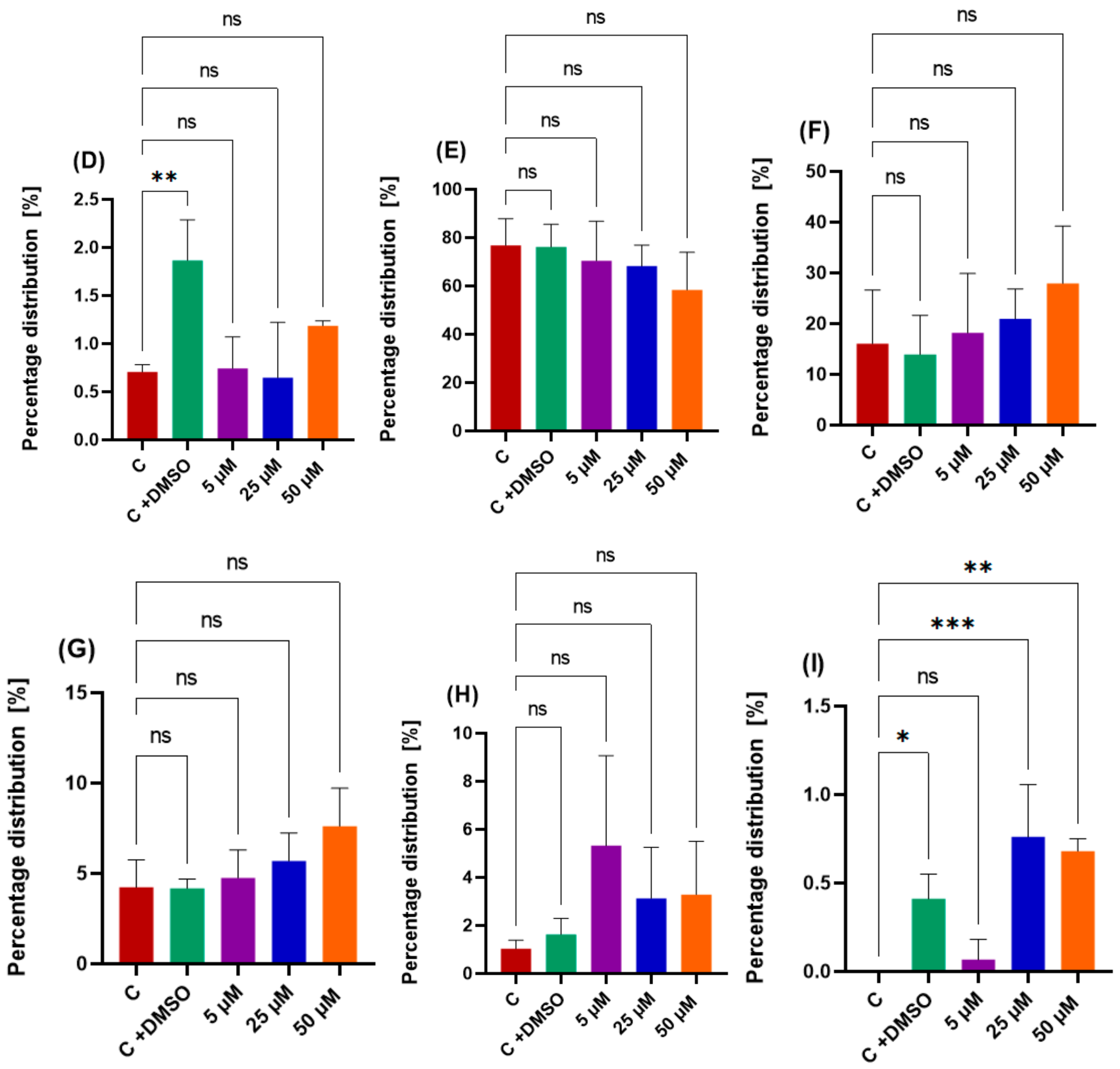

Figure S1. Percentage distribution of erythrocyte morphological forms induced by iodolactone **LV2**: (**A**) spherostomatocytes (SST(-4)), (**B**) second-order stomatocytes (ST2(-3)), (**C**) first-order stomatocytes (ST1(-2)), (**D**) discostomatocytes (DST(-1)), (**E**) discocytes (D(0)), (**F**) discoechinocytes (DE(1)), (**G**) echinocytes (E(2)), (**H**) spheroechinocytes (SE(3)) and (**I**) spherocytes (S(4)). Statistical analysis was performed using one-way ANOVA followed by Dunnett's post hoc test for comparisons with the control group. Statistical significance relative to the control group was denoted as follows: ns - not significant; * $p < 0.05$, ** $p < 0.01$, *** $p < 0.001$ and **** $p < 0.0001$.

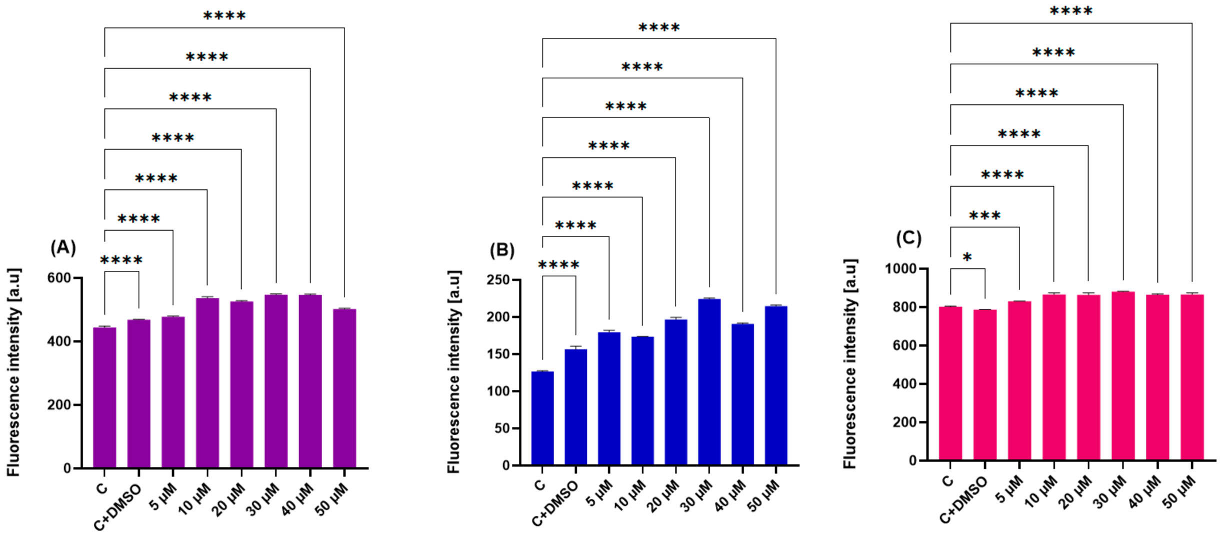

Figure S2. Concentration-dependent changes in MC540 fluorescence intensity in (**A**) LEMs, (**B**) RBCMs and (**C**) MCLMs following treatment with iodolactone **LV2**. Statistical analysis was performed using one-way ANOVA followed by Dunnett's post hoc test for comparisons with the control group. Statistical significance relative to the control group was denoted as follows: ns - not significant; * $p < 0.05$, ** $p < 0.01$, *** $p < 0.001$ and **** $p < 0.0001$.

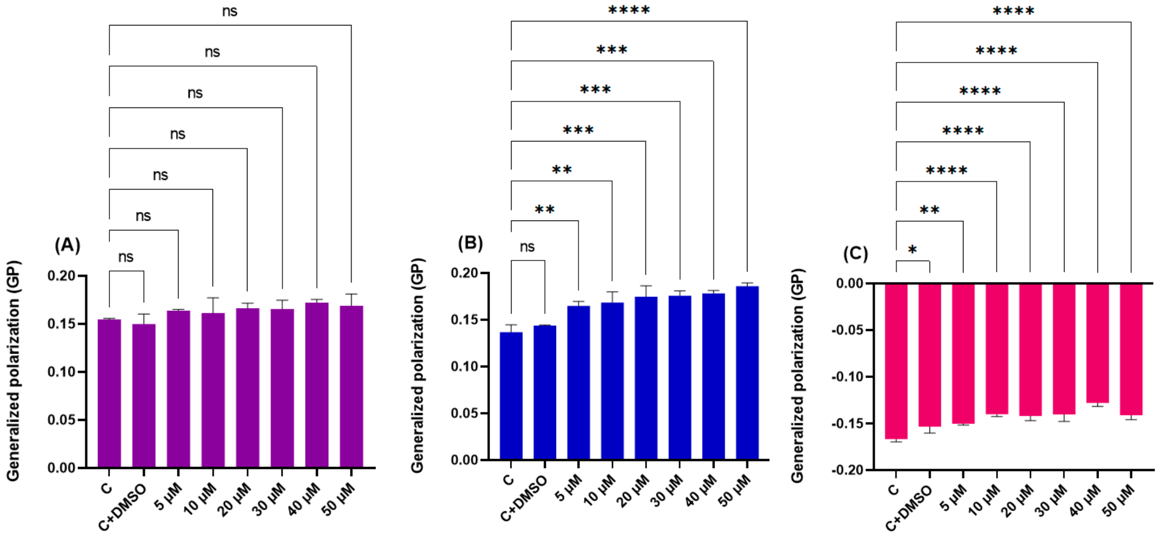

Figure S3. Changes in Laurdan generalized polarization (GP) in (**A**) LEMs, (**B**) RBCMs and (**C**) MCLMs following treatment with iodolactone **LV2**. Statistical analysis was performed using one-way ANOVA followed by Dunnett's post hoc test for comparisons with the control group. Statistical significance relative to the control group was denoted as follows: ns - not significant; * $p < 0.05$, ** $p < 0.01$, *** $p < 0.001$ and **** $p < 0.0001$.

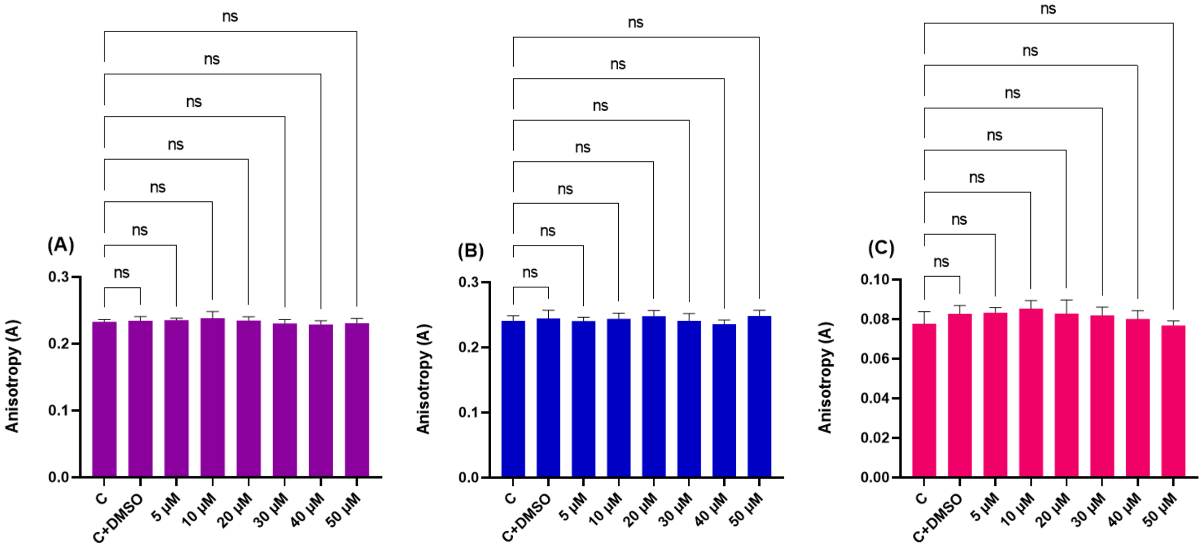

Figure S4. Changes in DPH anisotropy (A) in (**A**) LEMs, (**B**) RBCMs and (**C**) MCLMs following treatment with iodolactone **LV2**. Statistical analysis was performed using one-way ANOVA followed by Dunnett's post hoc test for comparisons with the control group. Statistical significance relative to the control group was denoted as follows: ns - not significant; * $p < 0.05$, ** $p < 0.01$, *** $p < 0.001$ and **** $p < 0.0001$.

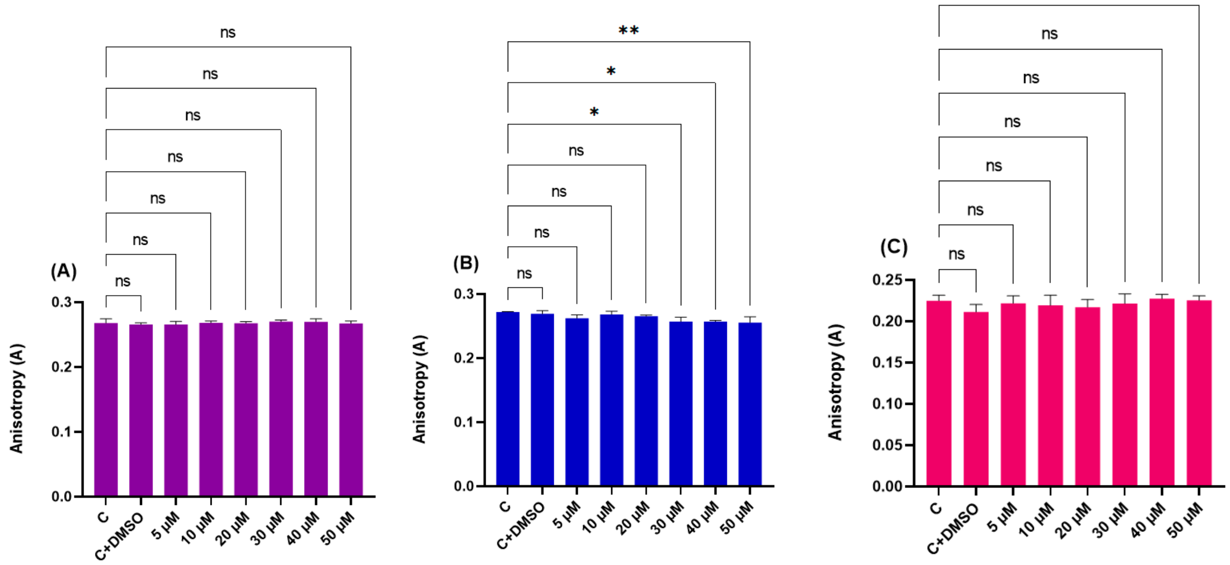

Figure S5. Changes in TMA-DPH anisotropy (A) in (**A**) LEMs, (**B**) RBCMs and (**C**) MCLMs following treatment with iodolactone **LV2**. Statistical analysis was performed using one-way ANOVA followed by Dunnett's post hoc test for comparisons with the control group. Statistical significance relative to the control group was denoted as follows: ns - not significant; * $p < 0.05$, ** $p < 0.01$, *** $p < 0.001$ and **** $p < 0.0001$.

1. *Chemicals and Reagents*

1.1. Free-Radical Scavenging Assays

DPPH• (2,2-diphenyl-1-picrylhydrazyl radical), ABTS•+ (2,2′-azobis(3-ethylbenzothiazoline-6-sulfonic acid cation radical), DMSO (biological grade), Trolox® (6-hydroxy-2,5,7,8-tetramethylchroman-2-carboxylic acid) and methanol (UHPLC purity) were purchased from Sigma-Aldrich® (Steinheim, Germany).

1.2. TBARS Assay

Phosphatidylcholine from egg yolk (EPC) was obtained from Lipid Products (Redhill, UK). The DMSO (dimethyl sulfoxide, biological grade) and Trolox® (6-hydroxy-2,5,7,8-tetramethylchroman-2-carboxylic acid) were purchased from Sigma-Aldrich® (Steinheim, Germany). Chloroform was purchased from Chempur (Piekary Śląskie, Poland).

1.3. *MTT Tests*

The RPMI-1640 (Roswell Park Memorial Institute), L-glutamine (L-Glu), streptomycin (Str), penicillin (Pen), FBS (Fetal Bovine Serum), DMSO (dimethyl sulfoxide, biological grade), trypsin, EDTA (Ethylenediaminetetraacetic Acid), HBSS (Hanks' Balanced Salt Solution), doxorubicin and MTT (3-(4,5-dimethylthiazol-2-yl)-2,5-diphenyltetrazolium bromide) were purchased from Sigma-Aldrich® (Steinheim, Germany). The DMEM (Dulbecco's Modified Eagle's Medium) was obtained from Thermo Fisher Scientific (Waltham, MA, USA). Isopropyl alcohol was purchased from Stanlab (Lublin, Poland).

1.4. Fluorometric Methods

Merocyanine 540 (MC540) and cholesterol were purchased from Sigma-Aldrich® (St Louis, MO, USA). PI (L-α-phosphatidylinositol sodium salt from *Glycine max*), SM (sphingomyelin), DMF (*N,N*-dimethylformamide) and *n*-butanol were purchased from Sigma-Aldrich® (Steinheim, Germany). Chloroform was purchased from Chempur (Piekary Śląskie, Poland).

The probes DPH (1,6-diphenyl-1,3,5-hexatriene), TMA-DPH (*N,N,N*-trimethyl-4-(6-phenyl-1,3,5-hexatrien-1-yl)phenylammonium *p*-toluenesulfonate) and Laurdan (6-dodecanoyl-2- dimethylamino naphthalene) were purchased from Molecular Probes (Eugene, OR, USA).

The POPC (1-palmitoyl-2-oleoylphosphatidylcholine), POPE (1-palmitoyl-2-oleoylphosphatidylethanolamine) and SOPS (1-stearoyl-2-oleoyl-*sn*-glicero-3-phospho-L-serine sodium salt) were purchased from Avanti Polar Lipids (Delfzijl, The Netherlands).
